# Enhanced non-viral gene delivery via calcium phosphate/DNA co-precipitates with low-voltage pulse electroporation in NK-92 cells for immunocellular therapy

**DOI:** 10.1063/5.0198191

**Published:** 2024-08-06

**Authors:** Che-Yung Kuan, I-Hsuan Yang, Chia-Ting Chang, Zhi-Yu Chen, Jhih-Ni Lin, Wei-Ting Kuo, Yu-Ying Lin, Andrew Yueh, Feng-Huei Lin

**Affiliations:** 1 Institute of Biomedical Engineering and Nanomedicine, National Health Research Institutes, No. 35, Keyan Road, Zhunan, Miaoli 35053, Taiwan; 2 Department of Biomedical Engineering, College of Medicine and College of Engineering, National Taiwan University, No. 49, Fanglan Rd., Taipei 10672, Taiwan; 3 PhD Program in Tissue Engineering and Regenerative Medicine, National Chung Hsing University, Taichung, Taiwan; 4 Institute of Biotechnology and Pharmaceutical Research, National Health Research Institutes, No. 35, Keyan Road, Zhunan, Miaoli 35053, Taiwan

## Abstract

Achieving high cell transfection efficiency is essential for various cell types in numerous disease applications. However, the efficient introduction of genes into natural killer (NK) cells remains a challenge. In this study, we proposed a design strategy for delivering exogenous genes into the NK cell line, NK-92, using a modified non-viral gene transfection method. Calcium phosphate/DNA nanoparticles (pDNA-CaP NPs) were prepared using co-precipitation methods and combined with low-voltage pulse electroporation to facilitate NK-92 transfection. The results demonstrated that the developed pDNA-CaP NPs exhibited a uniform diameter of approximately 393.9 nm, a DNA entrapment efficiency of 65.8%, and a loading capacity of 15.9%. Furthermore, at three days post-transfection, both the transfection efficiency and cell viability of NK-92 were significantly improved compared to standalone plasmid DNA (pDNA) electroporation or solely relying on the endocytosis pathway of pDNA-CaP NPs. This study provides valuable insights into a novel approach that combines calcium phosphate nanoparticles with low-voltage electroporation for gene delivery into NK-92 cells, offering potential advancements in cell therapy.

## I. INTRODUCTION

Natural killer (NK) cells are critical members of the innate immune system and act as the frontline defense against infections and malignant transformations.^1,2^ Their ability to swiftly respond to infected or aberrant cells without the need for prior sensitization makes them invaluable components of the immune system.^3–5^ Furthermore, NK cells have garnered significant attention in recent years owing to their potential in cancer immunotherapy. These cells possess a remarkable capacity to recognize and eliminate transformed and stressed cells in various tissues, including tumor cells.^6–8^

In addition to primary donor-derived NK cells, researchers have actively cultivated the NK cell line.^9–11^ One promising avenue for NK cell-based cancer immunotherapy involves the development of NK cell lines, such as NK-92, for adoptive cell transfer. Phase I clinical trials have demonstrated the safety and efficacy of NK-92 cell infusions, leading to clinical responses in some cancer patients.^12^ NK-92 cells possess a wide repertoire of activating receptors, including CD2 and CD56 adhesion molecules, and can lyse tumor cells *in vitro.*^13–15^ Owing to their extensive receptor repertoire and their capacity for interferon (IFN)-γ secretion and cytotoxicity, NK-92 cells are recognized as the most “NK cell-like” cell line, rendering them invaluable for clinical applications and research endeavors.^16^

To further enhance their therapeutic potential, NK-92 cells have undergone modifications to specifically target ligand expression on tumor cells, mirroring the principles of chimeric antigen receptor (CAR) T cells.^17,18^ Essentially, NK-92 cells have been transformed into off-the-shelf CAR-NK cells and meticulously cultured to exclusively recognize and eliminate tumor cells.^19–21^ The emergence of CAR-NK generation via NK-92 cells represents a promising frontier for cancer treatment, and the scope of its applications is expected to increase.^10,22^

Nevertheless, because of their unique attributes, NK cells exhibit high resistance to conventional transfection methods.^23^ Conventional gene delivery techniques are often limited by cost and efficiency constraints. The primary challenge associated with NK cell experimentation centers on their resistance to exogenous gene transfer. Adenoviral vectors, which rely on specific receptors absent from NK cells, have proven ineffective for this purpose.^23^ Although gene transfer reagents, lentiviral vectors, and retroviral vectors have played pivotal roles in generating clones,^24–26^ these virus-based transduction approaches come with limitations, including the potential to elicit immune responses, induce toxicity, and necessitate specialized equipment and expertise. Additionally, calcium phosphate transfection, a method that employs a mixture of calcium phosphate and genetic material to transport DNA or RNA into cells, is a straightforward and cost-effective technique widely adopted for cell transfection, particularly *in vitro.*^27,28^ However, the intrinsic resistance of NK cells to endocytosis often results in suboptimal outcomes.

In contrast, electroporation creates transient pores in the cell membrane using electric fields, enabling DNA to enter the cell.^29^ Although it has shown promise, electroporation can also damage cells, requiring careful control of voltage, duration, and pulse count to minimize harm or necessitating alterations to the electrolyte to enhance transfection efficiency.^30^ Other research has also utilized electroporation technology to enhance the efficacy of calcium phosphate-coated microneedles for delivering genes to the skin.^31^ Thus, identifying an optimal approach for gene transfer into NK cells is crucial to unlock their full potential in cancer immunotherapy.^32,33^

**FIG. 1.**
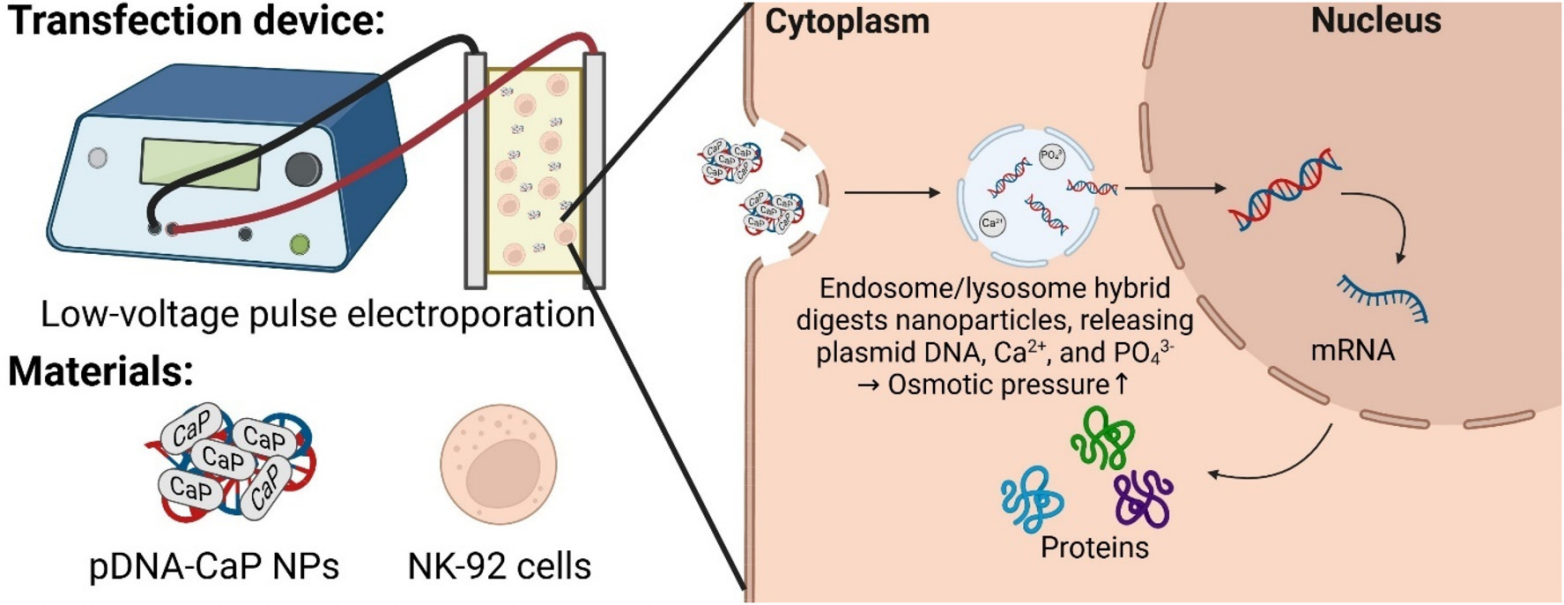
The scheme of pDNA CaP NPs combined with low-voltage pulse electroporation and its application for non-viral gene transfection in NK-92 cells.

In this study, a straightforward strategy that combines calcium phosphate transfection with modified electroporation to enhance the efficiency of gene transfer into NK cells was designed. By utilizing low-voltage pulses during electroporation and taking advantage of the rapid degradation of calcium phosphate within endosomes, we aimed to improve gene delivery while minimizing cellular damage. This groundbreaking approach holds the potential to revolutionize NK cell-based therapies, offering new avenues for the treatment of cancer and other diseases. Plasmid DNA (pDNA) carrying the humanized Renilla reniformis green fluorescent protein as a reporter gene was used to validate this hypothesis. This pDNA was co-precipitated with CaCl_2_ and Na_2_HPO_4_ to form pDNA-calcium phosphate nanoparticles (pDNA-CaP NPs). The developed pDNA-CaP NPs were characterized using agarose gel electrophoresis and NanoDrop spectrophotometry to evaluate their entrapment efficiency and loading capacity. Scanning electron microscopy (SEM), dynamic light scattering (DLS) spectroscopy, and x-ray powder diffraction (XRD) were used for microstructural observation, particle size measurement, zeta potential evaluation, and crystal structure determination. Cell viability and cytotoxicity were determined using water-soluble tetrazolium 1 (WST-1) and lactate dehydrogenase (LDH) assays. The NK-92 transfection efficiency and cell damage were evaluated using flow cytometry and propidium iodide (PI) staining. Further, the transfected NK-92 cells were observed using confocal laser microscopy for hrGFP expression. The experimental results were compared to those obtained from either standalone pDNA electroporation or exclusive use of the endocytic pathway of pDNA-CaP NPs. The overall design is illustrated in Fig. 1.

## II. RESULTS

### A. Agarose gel electrophoresis of pDNA-CaP NPs

**FIG. 2.**
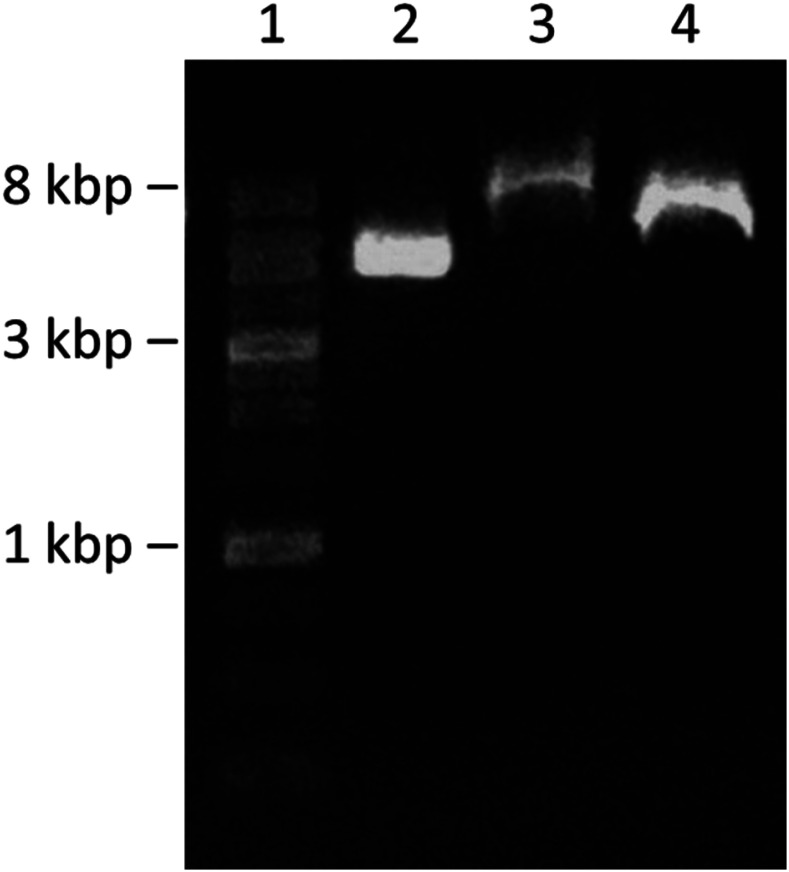
Agarose gel electrophoresis (1%) for detection of pDNA bands and pDNA-CaP NPs. Lane 1, 1 kb DNA size marker; lane 2, naked pDNA; lane 3, supernatant of the pDNA-CaP NPs group; and lane 4, released pDNA from the pDNA-CaP NPs via citrate buffer.

Agarose gel electrophoresis was used to investigate the successful entrapment of pDNA into CaP NPs, the pH-dependent dissolution of CaP nanoparticles, and the consequent release of pDNA (Fig. 2). A clear band around 5 kb appeared in the free pDNA group (lane 2). However, in lane 3, representing the supernatant after the co-precipitation of pDNA-CaP NPs, the bands decreased, indicating that most of the pDNA was successfully entrapped within the CaP NPs. The remaining non-entrapped pDNA formed the pDNA-calcium phosphate agglomerates, resulting in a higher molecular weight band in lane 3. The NPs were then dissolved in the citrate buffer to mimic the endosome microenvironment to release pDNA. In lane 4, although the band had a slightly higher molecular wright than that of lane 2, possibly due to partially undissolved NPs, the clear and intense band indicated that pDNA was successfully released from the pDNA-CaP NPs and did not degrade in the acidic microenvironment. A schematic illustration of the materials used for the agarose gel electrophoresis is shown in supplementary material Fig. S1.

However, in lane 3 of the supernatant after the co-precipitation of pDNA-CaP NPs, the bands decreased, indicating most of the pDNA were successfully entrapped within the CaP NPs. The resultant non-entrapped pDNA would form the pDNA-calcium phosphate agglomerates, resulting in a higher molecular weight band in lane 3.

### B. Morphology, particle size, and zeta potential evaluation of pDNA-CaP NPs

**FIG. 3.**
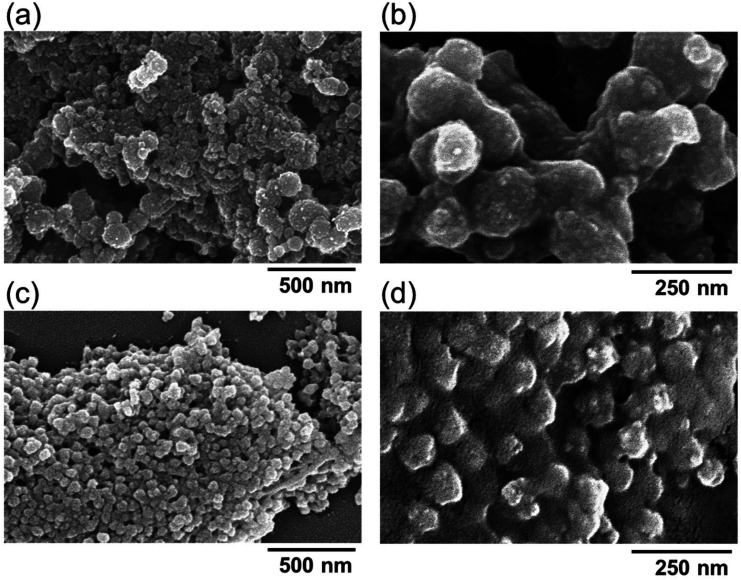
Morphology and microstructure of calcium phosphate nanoparticles analyzed via SEM. (a) Bare calcium phosphate nanoparticles were observed under 60 000× magnification and (b) under 140 000× magnification. (c) pDNA-CaP NPs were observed under 60 000× magnification and (d) under 140 000× magnification.

**FIG. 4.**
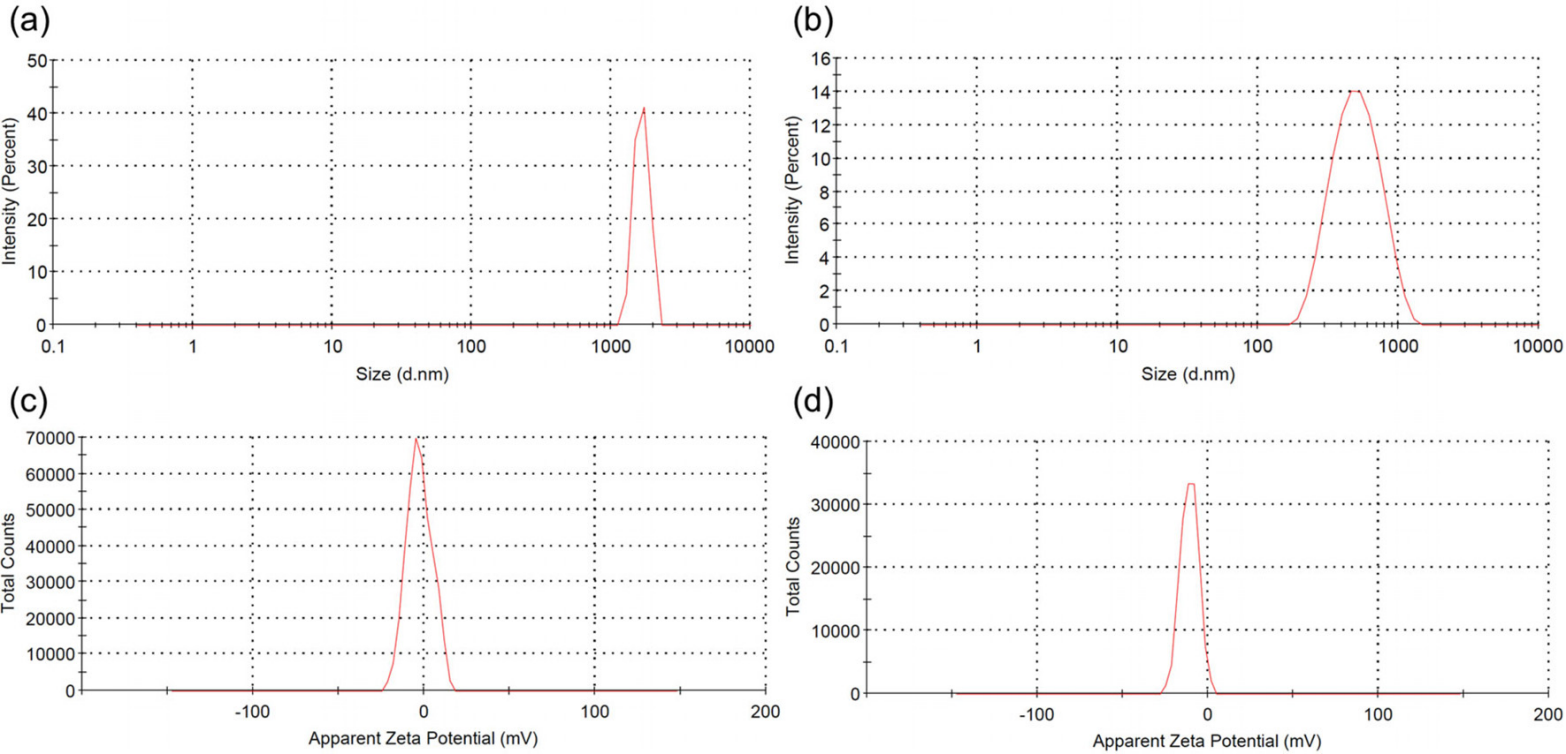
Particle size distribution and zeta potential spectra of the developed CaP NPs and pDNA-CaP NPs. (a) Particle size distribution of CaP NPs without pDNA loaded, (b) particle size distribution of pDNA-CaP NPs, (c) zeta potential spectrum of CaP NPs without pDNA loaded, and (d) zeta potential spectrum of pDNA-CaP NPs.

The morphology of the pDNA-CaP NPs was examined using SEM, as shown in Fig. 3. All grains exhibited a round morphology and were uniform with a diameter of ∼95 nm. The average hydrodynamic particle size and distribution of pDNA-CaP NPs were further determined using DLS, as shown in Figs. 4(a) and 4(b) and summarized in Table I. The average hydrodynamic particle size of pDNA-CaP NPs was 393.9 nm, whereas the polydispersity index (PDI) was 0.27, indicating that the particles were well dispersed in the solution [Fig. 4(b)]. The hydrodynamic particle size of bare CaP NPs without pDNA loaded was 3028.3 nm, and the PDI was 0.34 owing to aggregation [Fig. 4(a)]. During the synthesis of pDNA-CaP NPs, pDNA initially binds to calcium ions and disperses well in the solution.

**TABLE I.** Physicochemical characteristics, including particle size, zeta potential, polydispersity index, entrapment efficiency (EE), and loading capacity (LC), of pDNA-CaP NPs determined using DLS and NanoDrop.

Sample	*z*-average diameter^a^ (nm)	PDI^b^	Zeta potential (mV)	EE (%)	LC (%)
CaP NPs	3082.3 ± 419.1	0.34 ± 0.09	−2.75	⋯	⋯
pDNA-CaP NPs	393.9 ± 2.9	0.27 ± 0.01	−10.8	62.0 ± 13.4	15.9 ± 3.3

^a^ *z*-average diameter is the average diameter calculated based on the intensity of scattered light.

^b^ PDI = polydispersity index, a measure of the ratio between the weight-average particle diameter and the number-average particle diameter.

This helps to slow down the co-precipitation of CaP NPs, thereby reducing rapid particle aggregation and particle size. Moreover, compared to CaP NPs without loaded pDNA [Fig. 4(c)], the zeta potential of pDNA-CaP NPs increased to −10.8 mV [Fig. 4(d)] due to the inherent negative charge of pDNA, confirming the successful entrapment of pDNA within the pDNA-CaP NPs.

### C. Entrapment efficiency and loading capacity of pDNA-CaP NPs

To confirm that pDNA was successfully entrapped into the CaP NPs, the pDNA-CaP NPs were collected through centrifugation at 20 000 rpm for 1 h. Unentrapped free pDNA was suspended in the supernatant, and its concentration was measured using the NanoDrop. Entrapment efficiency was calculated using the following formula:

$$\text{EE}\;(\%)=\frac{m\text{ass}\;\text{of}\;\text{pDNA}\;\text{used}\;\text{in}\;\text{fo}r\text{mulation}-m\text{ass}\;\text{of}\;\text{unentrappd}\;\text{pDNA}\;\text{in}\;\text{supernatant}}{m\text{ass}\;\text{of}\;\text{pDNA}\;\text{used}\;\text{in}\;\text{foumulation}}\times 100\%.$$

The mass of the pDNA-CaP NPs was measured, and the loading capacity (LC) was calculated using the following formula:

$$\text{LC}\;(\%)=\frac{m\text{ass}\;\text{of}\;\text{pDNA}\;\text{in}\;\text{pDNA}-\text{CaP}\;\text{NPs}}{m\text{ass}\;\text{of}\;\text{pDNA}-\text{CaP}\;\text{NPs}}\times 100\%.$$

The entrapped efficiency of pDNA-CaP NPs was calculated to be 65.8%, and the loading capacity was calculated to be 15.9%, as summarized in Table I.

### D. Crystal structure of pDNA-CaP NPs

**FIG. 5.**
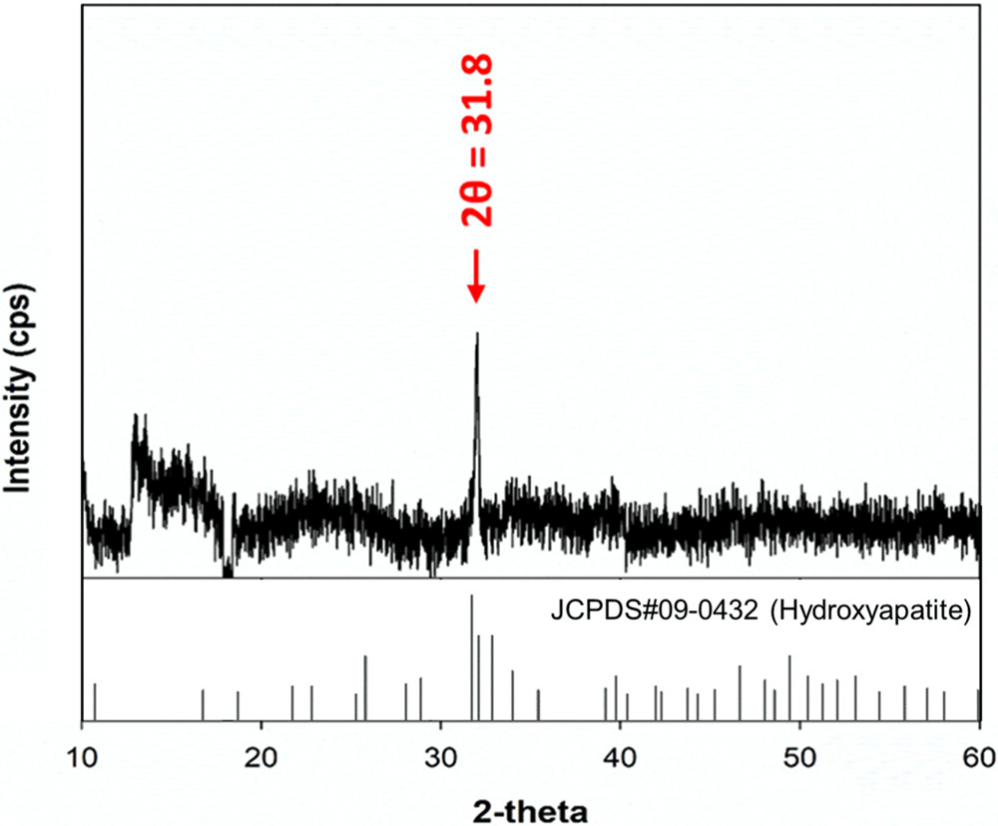
XRD patterns recorded on pDNA-CaP NPs and standard pattern of hydroxyapatite (JCPDS No. 09-0432). The characteristic peak at 31.8° confirmed that the sample exhibited typical hydroxyapatite crystals (2 1 1).

XRD was employed to examine the phase and crystalline characteristics of pDNA-CaP NPs. As shown in Fig. 5, the appearance of a distinctive peak at 2θ = 31.8° indicated the presence of the hydroxyapatite phase, represented by the crystallographic planes (2 1 1), fully matched the standard XRD pattern of the hydroxyapatite (JCPDS Card No. 09-0432), confirming the formation of typical hydroxyapatite crystals within the nanoparticles. Numerous additional peaks attributed to hydroxyapatite were not very remarkable owing to the low signal-to-noise. The absence of other peaks in the XRD pattern, specifically those indicating the presence of secondary phases of CaP, such as monetite with planes (1 2 0) at 2θ = 30.24° or β-tricalcium phosphate (β-TCP) with planes (0 2 1 0) at 30.11°, further validated the purity and specific crystalline structure of the synthesized nanoparticles.^34,35^ This level of purity and structural specificity is crucial for the intended biomedical applications of these nanoparticles, as it can significantly influence their biological interactions and efficacy.

The Schiff bases between 1631.5 and 1640.9 cm^−1^ attributed to the Cdouble bondN stretch characteristics of the imino group were not very remarkable, owing to the strong absorption of the carboxyl group and the low concentration of laminin.^34^

The XRD pattern of NZ-PCNF also revealed the characteristic peak of zeolite with a slightly reduced crystallinity owing to the disordered graphitic structure. This result indicates that the NZ-PCNF have a graphitic carbon structure and that the crystalline zeolite structure does not change appreciably during the carbonization process.

### E. Cytotoxicity of pDNA-CaP NPs

**FIG. 6.**
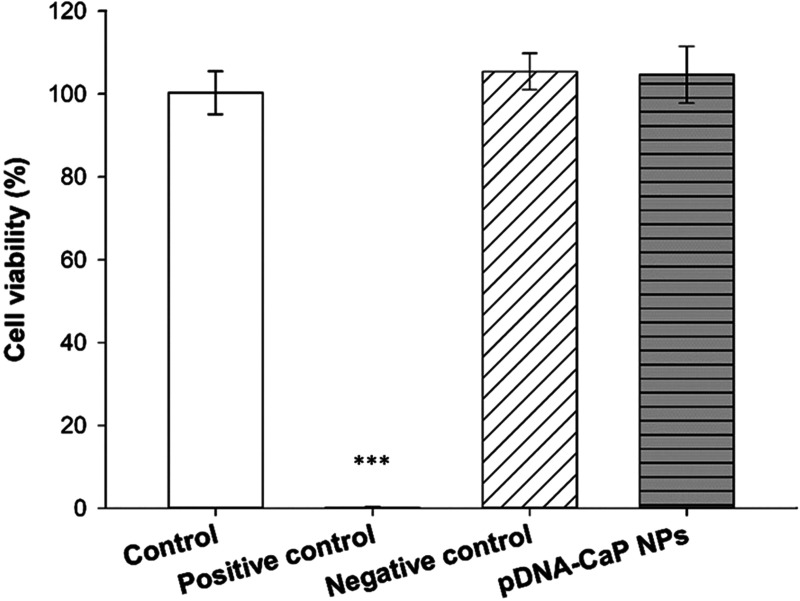
Cell viability of pDNA-CaP NPs in L929 cells by WST-1 assay, following the ISO 10993-5 guideline. After 24 h of incubation, cell viability was determined using the WST-1 assay. The control group consisted of cells cultured in the normal medium. ZDEC extract was employed as the positive control group, while Al_2_O_3_ extract served as the negative control group. (*p* < 0.001, ^***^ compared to control group by one-way ANOVA.)

**FIG. 7.**
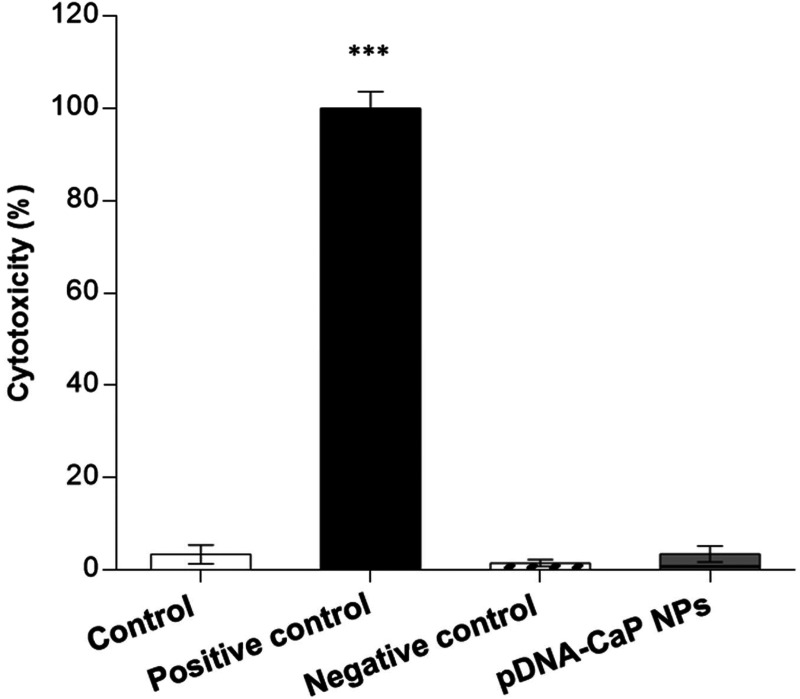
Cell death rate of pDNA-CaP NPs in L929 cells by LDH assay, following the ISO 10993-5 guideline. The results are shown in terms of cytotoxicity. After 24 h of incubation, cytotoxicity was determined using the LDH assay. The control group consisted of cells cultured in the normal medium. ZDEC extract was employed as the positive control group, while Al_2_O_3_ extract served as the negative control group. (*p* < 0.001, ^***^ compared to control group by one-way ANOVA.)

To confirm the safety of the developed pDNA-CaP NPs as gene delivery carriers, their cytotoxicity was evaluated using WST-1 and LDH assays according to the ISO 10993-5 standard. The viability of pDNA-CaP NPs in the target cells is shown in Fig. 6. The normal cell culture medium served as the control, and the extracts of ZDEC and Al_2_O_3_ served as positive and negative controls, respectively. The cell viability of the pDNA-CaP NPs was greater than 80%, indicating that the developed NPs were not cytotoxic to the target cells. However, if the material induces cell proliferation, leading to an increased number of cells, this could also result in higher cell viability outcomes. Therefore, evaluating cell death is also necessary to provide complementary insights into the effects of the material on cells. The LDH assay was used to evaluate the rate of cell death, as shown in Fig. 7. The cell death rate of the developed pDNA-CaP NPs was less than 5 %, indicating that the developed pDNA-CaP NPs showed good biocompatibility.

### F. Transfection efficiency

**FIG. 8.**
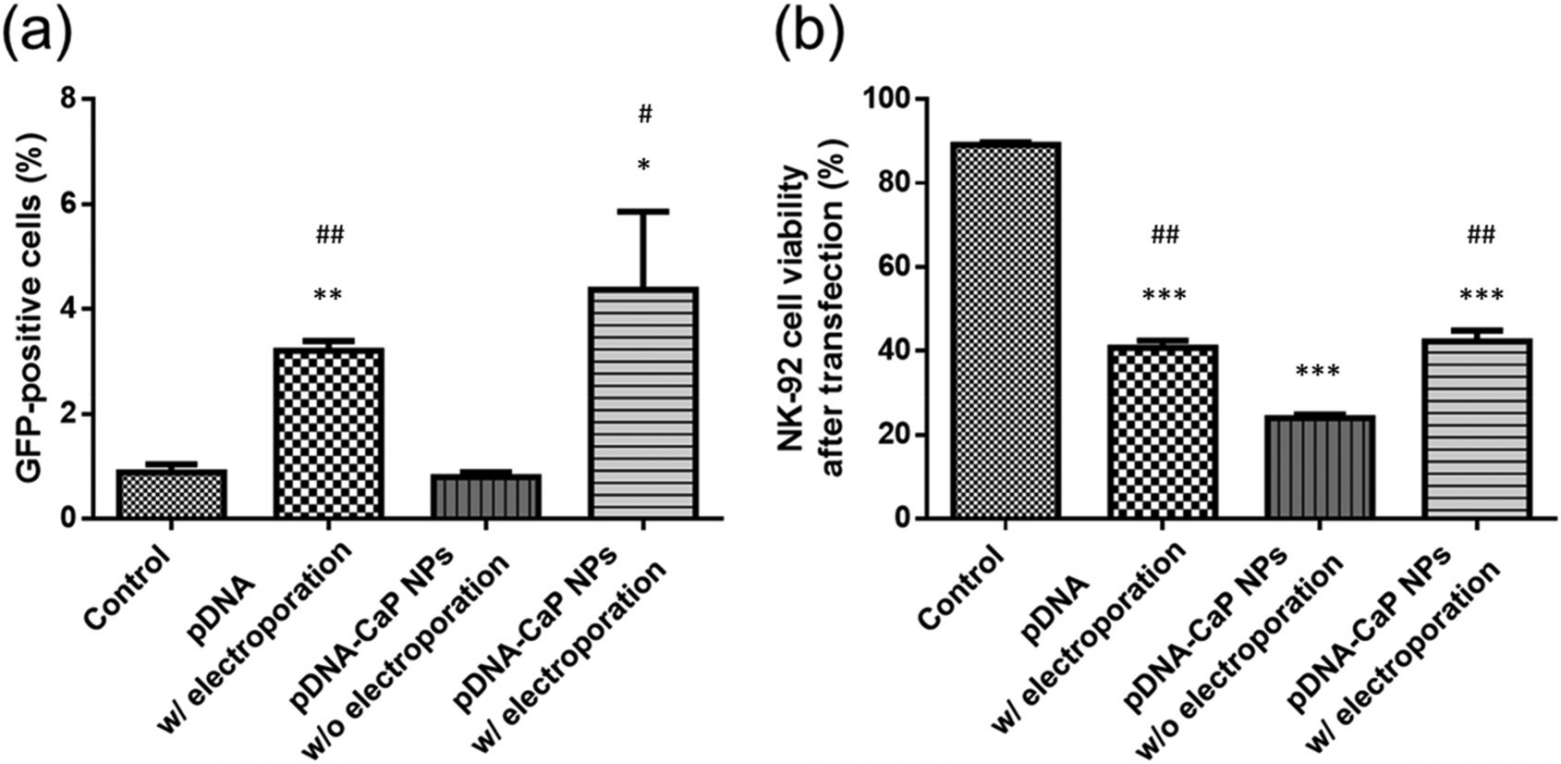
Transfection efficiency and NK-92 cell viability after transfection by different methods. (a) GFP-positive cells were quantified by flow cytometry. (b) Cell viability was determined using propidium iodide (PI) staining viability dye and quantified by flow cytometry. (*p* < 0.05, ^*^; *p* < 0.01, ^**^; *p* < 0.001, ^***^, compared to the control group; *p* < 0.05, #; *p* < 0.01, ##, compared to the pDNA-CaP NPs w/o electroporation group by one-way ANOVA.)

NK-92 cells were collected and analyzed for the number of successfully transfected green fluorescent cells and cell viability through flow cytometry (Fig. 8). In the control group, NK-92 cells showed no green fluorescence and cell viability was nearly 90%. When NK-92 cells were co-cultured with the developed pDNA-CaP NPs without assisted electroporation, owing to NK-92's inherent resistance to cellular uptake, the transfection efficiency was not significantly different from that of the control group, and it caused a certain degree of cell death, with a cell viability of only about 24.0%. Using electroporation alone to transfect free pDNA resulted in a transfection success rate of approximately 3.2%. Nevertheless, owing to factors, such as voltage, duration, and the high sensitivity of NK-92 cells to the environment, cell viability was about 40.7%. These limitations could potentially hinder their application in cancer treatment.

In contrast, when NK-92 cells were exposed to a combination of pDNA-CaP NPs and modified electroporation, using lower-voltage pulses, the uptake of pDNA-CaP NPs by the cells significantly increased. Additionally, CaP NPs could possibly rapidly degrade within the acidic endosomal environment, leading to an increase in ion concentration and osmotic pressure. This ultimately caused endosome rupture and facilitated the escape of pDNA from the endosome, resulting in successful transfection. The results showed a transfection efficiency of approximately 4.4%, accompanied by an increase in cell viability to 42.3%, compared to using pDNA-CaP NPs alone, effectively mitigating the damage to the NK-92 cells. Furthermore, transfected cells were observed using confocal microscopy (supplementary material Fig. S2), and the results were consistent with the flow cytometry data. The group combining pDNA-CaP NPs with low-voltage pulse electroporation exhibited the highest cell transfection efficiency. Additionally, a lipid-based transfection reagent, Lipofectamine 2000, was also utilized for comparison of the cell transfection efficiency and viability, as summarized in Table II.

**TABLE II.** Summary of the transfection efficiency and cell viability of NK-92 cells via flow cytometry.

Flow cytometry	hrGFP (%)	EthD-1 (%)
Control	0.07 ± 0.06	10.86 ± 1.27
pDNA w/electroporation	7.44 ± 0.21	59.23 ± 4.12
pDNA-CaP NPs w/o electroporation	8.33 ± 0.23	76.00 ± 2.01
pDNA-CaP NPs w/electroporation	1.50 ± 0.30	57.71 ± 6.38
Lipofectamine 2000	19.40 ± 0.59	65.04 ± 4.21

## III. DISCUSSION

In recent research, CAR-T cell therapy targeting the CD19 antigen and B-cell maturation antigen (BCMA) has emerged as a promising treatment option for blood cancers such as lymphoma, leukemia, and myeloma, with six CAR-T cell products receiving approval from the US FDA.^36–39^ However, the use of CAR-T cells has limitations, including off-target effects and cytokine storms.^40,41^ Moreover, CAR-T cell therapy has not yet achieved success in patients with solid tumors. In addition, obtaining a sufficient quantity of autologous T cells and proliferating them within a clinical setting presents significant limitations, making it challenging to obtain the required number of CAR-T cells within a short timeframe.^42^ Utilizing allogeneic T cells from a different donor may overcome the challenge of a limited cell source; however, this approach carries the risk of severe graft-vs-host disease (GVHD).^43^

Conversely, CAR-NK-92 cells exhibit high cytotoxicity, can be harvested as a homogenous cell population, and can be produced in large quantities within a short timeframe. In comparison with CAR-T cells, CAR-NK-92 cells have a shorter *in vivo* survival period after activation, which mitigates off-target effects.^44^ However, owing to the inherent difficulty of successfully transfecting genes into NK-92 cells, research on non-viral vectors for NK-92 cells is still limited. Previous studies have used electroporation to introduce genes into NK-92 cells; however, increased cell membrane permeability and cell lysis often result in a high cell death rate.^32,33^ Therefore, researchers have focused on adjusting the electroporation medium to repeatedly and transiently deliver exogenous genes into NK-92 cells.^23^

In addition, CaP NPs possess pH-responsive dissolution properties. Upon endocytosis, CaP NPs swiftly degrade into Ca^2+^ and PO_4_^3−^ within the acidic environment of endosomes (pH 5).^45^ This elevated osmotic pressure within endosomes causes water to enter, ultimately rupturing the endosome membrane.^46^ This process facilitates the rapid escape of pDNA from the endosome into the cytoplasm, preventing pDNA from being transported into lysosomes and degraded by enzymes.^47^ Previous research has used low-intensity pulsed ultrasound to reduce cell membrane barriers and increase cell membrane permeability. Combining CaP NPs with ultrasound has achieved more efficient transfection and minimized cell damage compared to using ultrasound or CaP NPs alone.^48^

Currently, many researchers are actively seeking to perform gene editing on immune cells through non-viral transfection methods. Bulk gene electroporation methods are the simplest approach, involving the application of high voltages to generate transient pores on the cell membrane, achieving transfection efficiency of approximately 19%–50%.^49–51^ Factors, such as the size of DNA fragments, also influence the efficiency of DNA transfection. Additionally, some researchers have used manganese dioxide nanoparticles to deliver siRNA to NK-92 cells for the restoration of NK cell function.^52^ However, there is little research on the use of CaP NPs for transfection into NK-92 cells. In this study, we employed hrGFP as a reporter gene combined with low-voltage pulse electroporation to assess its suitability for non-viral transfection in NK-92 cells. Previous research has shown that the optimal cellular uptake particle size ranges from 50 to 500 nm.^53,54^ According to SEM and DLS analyses, the average hydrodynamic particle size of pDNA-CaP NPs was 393.9 ± 2.9 nm, making it suitable for cellular uptake (Figs. 3 and 4, and Table I). The XRD results confirmed the formation of hydroxyapatite crystal structures in the pDNA-CaP NPs synthesized in this study (Fig. 5), which was consistent with previous co-precipitation studies.^35^ This confirms that the process developed in this study can rapidly and uniformly synthesize the required pDNA-CaP NPs. During the optimization of the best entrapment efficiency and loading capacity, it should be noted that reducing the concentration of added pDNA can significantly enhance entrapment efficiency to 95%, although it may decrease loading capacity. Conversely, increasing the concentration of DNA can improve loading capacity, but the DNA molecules may aggregate during the coating process due to electrostatic interactions, leading to reduced entrapment efficiency. To strike a balance between these factors, this study optimized the parameters, achieving an entrapment efficiency of 65.8% and a loading capacity of 15.9%. According to the literature, a loading capacity between 10% and 20% is generally considered acceptable.^55–57^ The developed pDNA-CaP NPs showed no effect on cell viability or cytotoxicity in the mammalian cells (Figs. 6 and 7).

Fluorescence microscopy and flow cytometry revealed that both bare pDNA with electroporation and pDNA-CaP NPs alone had limited success in cell transfection and viability. Conversely, when pDNA-CaP NPs were combined with low-voltage pulsed electroporation, cell membrane permeability increased, helping pDNA-CaP NPs enter NK-92 cells (Fig. 8). It is noteworthy that although Lipofectamine 2000 exhibited better transfection efficiency, it resulted in decreased survivability of NK-92 cells (Table II). While there is no statistical difference between the transfection efficiency of pDNA-CaP NPs combined with electroporation and that of bare pDNA with electroporation, pDNA-CaP NPs combined with electroporation achieved the highest transfection efficiency. It is speculated that pDNA entrapped within the CaP NPs can remain intact and stable in the physiological environment, reduce NK-92 cells' inherent resistance to cellular uptake, and minimize the harm caused by electroporation. Thus, further evaluation should analyze factors such as the fragment size of pDNA, the ratio of pDNA-CaP NPs to the electrolyte, and electric pulse parameters, to optimize the transfection efficiency and cell viability.

In this study, pDNA was fully entrapped within the CaP NPs and escaped from the endosome in an intact DNA structure when exposed to an acidic environment (Fig. 2). This approach improved the transfection efficiency and cell viability compared to using bare pDNA alone.

Although there is no statistical difference between the pDNA-CaP NPs combined with electroporation and bare pDNA with electroporation, pDNA-CaP NPs combined with electroporation has the highest transfection efficiency. It is speculated that pDNA entrapped within the CaP NPs can maintain intact and stable in the physiological environment, reduce the NK-92's inherent resistance to cellular uptake, and minimize harm of electroporation. Thus, the further evaluation should be analyzed, such as the fragment size of pDNA, ratio of pDNA-CaP NPs to the electrolyte, and electric pulse parameters, to optimize the transfection efficiency and cell viability.

In this study, we demonstrated that the combination of pDNA-CaP NPs and low-voltage pulsed electroporation is a promising gene delivery system that can be applied to NK-92 gene modification. The efficacy of this method was compared to that of the traditional approach of applying pDNA alone for electroporation or solely using endocytosis of pDNA-CaP NPs. Furthermore, the developed pDNA-CaP NPs can be prepared in just 30 min and scaled up for production to meet clinical demands. However, expanding cell numbers to the required level for cancer treatment (10^9^–10^10^ cells) remains a challenge,^21,58^ and further evaluation is needed to determine the efficiency of applying the developed gene transfection system to large-scale production of genetically modified NK-92 cells in the future.

## IV. CONCLUSION

In this study, we successfully synthesized pDNA-CaP NPs using a co-precipitation method. The prepared nanoparticles exhibited a narrow particle size distribution and excellent dispersion in an aqueous solution. Moreover, the developed pDNA-CaP NPs served as efficient gene carriers without cytotoxicity, displaying a high DNA entrapped efficiency of 65.8% and a loading capacity of 15.9%. The combination of pDNA-CaP NPs with low-voltage pulse electroporation made NK-92 cells more receptive to transfection, resulting in higher survival rates. These findings suggest that the developed pDNA-CaP NPs, in conjunction with low-voltage electroporation, hold potential as a non-viral gene delivery method for NK-92 cells and may find wide-ranging applications in cancer immunotherapy.

## V. METHODS

### A. Materials

NK-92 cells were purchased from ATCC (Manassas, VA, USA). Calcium chloride, 2× HEPES buffered saline, and minimum essential medium eagle alpha modification (α-MEM) were purchased from Sigma-Aldrich (St. Louis, MO, USA). Fetal bovine serum (FBS) was purchased from HyClone (Logan, Utah, USA). Plasmid pIRES-hrGFP-1a, which encodes the hrGFP reporter gene and the cytomegalovirus promoter, was obtained from Agilent Technologies (Santa Clara, CA, USA). A plasmid DNA purification kit was obtained from Qiagen (Chatsworth, CA, USA). Finally, the cell proliferation reagent WST-1 and LDH cytotoxicity detection kit were purchased from Roche (Basel, Switzerland).

### B. Amplification and purification of DNA plasmid

The hrGFP plasmid (pIRES-hrGFP-1a, 5 kb) was used to prepare pDNA-CaP NPs and monitor transgene expression. The hrGFP plasmid was purified using the Qiagen plasmid kit according to the manufacturer's instructions and then resuspended in distilled water. pDNA integrity was determined through 1% agarose gel electrophoresis, and DNA concentration was measured using a NanoDrop spectrophotometer (Nano-100, CLUBIO, Taiwan) at 260 nm.

### C. Preparation of pDNA-CaP nanoparticles

The pDNA-CaP NPs were prepared using co-precipitation methods. First, 7.5 *μ*l of 2.5 M CaCl_2_ were mixed with 25 *μ*g of pDNA and brought to a total of 75 *μ*l with distilled H_2_O. The resultant solution was then dropwise added to the 75 *μ*l 2× HEPES Buffered Saline (pH 7.05), which contains 50 mM HEPES, 280 mM NaCl, and 1.5 mM Na_2_HPO_4_, with gentle agitation. After vortexing for 4 s, the resultant solution was allowed to stand for 20 min to allow co-precipitation to prepare the pDNA-CaP NPs.

### D. Electrophoresis of pDNA-CaP NPs

To assess the success of pDNA co-precipitation in the CaP NPs, the unentrapped pDNA supernatant layer after preparation of pDNA-CaP NPs and the pDNA-CaP NPs after dissolution in citrate buffer (pH 5.0) were stained with 1× fluorescent tracking dyes (Novel Juice PLUS, GeneDireX, Taiwan) and visualized using 1% agarose gel electrophoresis (30 min at 100 V and 3000 mA) under ultraviolet light. The 1 k DNA ladder (DM3100, SMOBIO) was used as a reference for the desired positive 5 kb of pDNA.

### E. Morphology observation of pDNA-CaP NPs

After the co-precipitation of pDNA-CaP NPs, the NPs were collected and washed twice with distilled H_2_O. The collected NPs were then dried in an oven, mounted on an Al stage, and coated with a gold film via ion sputtering. The pDNA-CaP NPs were examined under an SEM (S-4700, Hitachi, Japan) at 10 kV for microstructural examination.

### F. Particle size and zeta potential analysis of pDNA-CaP NPs

The particle size and zeta potential of the developed pDNA-CaP NPs were evaluated using a Nano-ZS (Malvern Instruments, UK) based on dynamic light scattering measurements and laser Doppler electrophoresis, respectively. The developed pDNA-CaP NPs were suspended in distilled H_2_O. The particle size was determined at 25 °C with a 90° scattering angle based on the internal setting. The zeta potential was measured using an aqueous flow cell in the automatic mode at 25 °C.

### G. Entrapment efficiency and loading capacity of pDNA-CaP NPs

The entrapment efficiency (EE) of the pDNA-CaP NPs was determined using the NanoDrop. After the co-precipitation of pDNA-CaP NPs, the NPs were collected by centrifugation at 20 000 g for 1 h. The unentrapped pDNA was suspended in the supernatant and quantified using the NanoDrop spectrophotometer at 260 nm.

### H. Crystal structure of pDNA-CaP NPs

The crystal structure of the synthesized pDNA-CaP NPs was determined using an x-ray powder diffractometer (Rigaku Miniflex-II, Japan) with Cu Kα radiation (λ  =  1.5406 Å), operated at 30 kV and 15 mA using a scan rate of 2.0°/min (2θ  =  10°–60°).

### I. Cytotoxicity of pDNA-CaP NPs

The cytotoxicity of pDNA-CaP NPs was evaluated using the WST-1 and LDH assays. For the WST-1 assay, L929 cells were seeded in a 96-well plate at a density of 5000 cells/well for 24 h. The α-MEM medium was used to extract pDNA-CaP NPs (0.2 g/ml) for the experimental group. Zinc diethyldithiocarbamate (ZEDC) and Al_2_O_3_ were extracted for 24 h at a concentration of 0.2 g/ml as a positive control group and negative group, respectively. The medium of the pre-seeded 96-well plate was then changed to extract for another 24 h. Cell viability was evaluated using the WST-1 kit with a plate reader (Enspire, PerkinElmer, USA) to detect the absorbance wavelength at 450 nm. For the LDH assay, the cells and extracts were treated as previously described. The cell death rate was determined using the LDH kit with a plate reader to detect the absorbance wavelength at 490 nm.

### J. NK-92 transfection

Plasmid DNA electroporation was performed using the Neon^®^ Transfection System (MPK5000, Invitrogen, USA). NK-92 cells were harvested by centrifugation at 1000 rpm for 5 min. The pellet was resuspended in 10 ml PBS and centrifuged at 1000 rpm for 5 min to remove the supernatant. The washed pellet was resuspended in Resuspension Buffer T (Invitrogen, USA). 2 × 10^5^ cells were transferred to a sterile 1.5 ml centrifuge tube, brought to a final cell suspension volume of 10 *μ*l, and mixed with pDNA-CaP NPs or free pDNA. The electroporation parameters were 1300 V, 10 ms, and three pulses. The electroporated NK-92MI cells were cultured in 500 *μ*l of culture medium without antibiotics. GFP-positive cells were identified on day 3 using a flow cytometer (Attune NxT, Invitrogen, USA) and confocal microscope (LSM 900, Zeiss, Germany).

### K. Statistics

The results are presented as mean and standard deviation, and a minimum of three independent measurements were conducted. One-way analysis of variance (ANOVA) with Tukey's multiple comparison test and Student's t-test were used for all statistical analyses. Statistical significance was determined at a p-value less than 0.05, denoted as follows: *p* < 0.05, ^*^; *p* < 0.01, ^**^; *p* < 0.001, ^***^.

## SUPPLEMENTARY MATERIAL

See the supplementary material for the materials used in agarose gel electrophoresis and NK-92 cells after different transfection methods, in Figs. S1 and S2, respectively.

## Data Availability

The data that support the findings of this study are available from the corresponding authors upon reasonable request.
